# Correlation between Genetic Variations and Serum Level of Interleukin 28B with Virus Genotypes and Disease Progression in Chronic Hepatitis C Virus Infection

**DOI:** 10.1155/2015/768470

**Published:** 2015-02-25

**Authors:** Ahmed Al-Qahtani, Mashael Al-Anazi, Ayman A. Abdo, Faisal M. Sanai, Waleed Al-Hamoudi, Khalid A. Alswat, Hamad I. Al-Ashgar, Mohammed Q. Khan, Ali Albenmousa, Nisreen Khalaf, Nisha Viswan, Mohammed N. Al-Ahdal

**Affiliations:** ^1^Department of Infection and Immunity, Research Center, King Faisal Specialist Hospital & Research Center, Riyadh, Saudi Arabia; ^2^Liver Disease Research Center, King Saud University, Riyadh, Saudi Arabia; ^3^Section of Gastroenterology, Department of Medicine, College of Medicine, King Saud University, Riyadh, Saudi Arabia; ^4^Gastroenterology Unit, Department of Medicine, King Abdulaziz Medical City, Jeddah, Saudi Arabia; ^5^Gastroenterology Unit, Department of Medicine, King Faisal Specialist Hospital & Research Center, Riyadh, Saudi Arabia; ^6^Department of Gastroenterology, Prince Sultan Medical Military City, Riyadh, Saudi Arabia

## Abstract

Recent studies have demonstrated that polymorphisms near the interleukin-28B (IL-28B) gene could predict the response to Peg-IFN-a/RBV combination therapy in HCV-infected patients. The aim of the study was to correlate the serum level of IL28B in HCV-infected patients with virus genotype/subgenotype and disease progression. IL28B serum level was detected and variations at five single nucleotide polymorphisms (SNPs) in IL28B gene region were genotyped and analyzed. The variation of IL28B genetic polymorphisms was found to be strongly associated with HCV infection when healthy control group was compared to HCV-infected patients with all *P* values <0.0001. Functional analysis revealed that subjects carrying rs8099917-GG genotype had higher serum level of IL28B than those with GT or TT genotypes (*P* = 0.04). Also, patients who were presented with cirrhosis (Cirr) only or with cirrhosis plus hepatocellular carcinoma (Cirr+HCC) had higher levels of serum IL28B when compared to chronic HCV-infected patients (*P* = 0.005 and 0.003, resp.). No significant association was found when serum levels of IL28B were compared to virus genotypes/subgenotypes. This study indicates that variation at SNP rs8099917 could predict the serum levels of IL28B in HCV-infected patients. Furthermore, IL28B serum level may serve as a useful marker for the development of HCV-associated sequelae.

## 1. Introduction

Approximately, 170 to 180 million people (~3% of the world population) are estimated to be infected with hepatitis C virus (HCV) [1]. Prolonged and persistent HCV infection may lead to cirrhosis and hepatocellular carcinoma (HCC) [2, 3]. Up until recently, the treatment of chronic HCV infection involved a 24- or 48-week course of pegylated interferon-alpha (PEG-IFN*α*) in combination with ribavirin (RBV) [4, 5]. Unfortunately, the efficacy of this treatment is limited, with only half of the patients are able to achieve a sustained virological response (SVR), particularly in patients with genotype 1 [4, 6]. More recently, newer and more potent antiviral agents have been approved with higher efficacy and tolerability but burdened with a vastly higher cost. Although PEG-IFN*α*/RBV therapy is not first line therapy nowadays in the majority of clinical scenarios it is still recommended in triple therapy combination regimens in many patients to shorten the duration of therapy. In addition, in many countries worldwide, interferon based therapy is still used since the newer, more potent antiviral agents are not routinely available yet because of the associated high cost (http://www.hcvguidelines.org/) [7].

With the recent advent of genome-wide association studies, three major studies have reported the significance of several single nucleotide polymorphisms (SNPs) in the vicinity of interleukin-28B (IL28B) gene, capable of predicting the response outcome of combination therapy in genotype-1 HCV infected patients. Ge et al. (2009) found that the SNP, rs12979860, which is 3 kb upstream of* IL28B* gene, was significantly associated with SVR in genotype-1 HCV-infected patients undergoing 48 weeks of PEG-IFN*α* plus RBV treatment [8]. In a study conducted on Japanese patients infected with HCV genotype-1, Tanaka et al. (2009) found additional SNPs, near IL28B gene, which were strongly associated with null virological response (NVR) and SVR, with rs8099917 being the most significant [9]. A similar study conducted on Australian patients, also concluded a strong association of rs8099917 with SVR in individuals infected with genotype-1 HCV and undergoing combination therapy [10]. Furthermore, a study conducted by Thomas et al. (2009) reported that variations in rs12979860 could play a pivotal role in the spontaneous, natural clearance of HCV [11]. Thus, much effort is being put in order to determine the predictive power of the genetic polymorphisms around the IL28B gene in relation to SVR, before it can be implemented into the current treatment therapy.

IL28B belongs to the cluster of interferon-*λ* (IFN-*λ*) genes that belong to the type-III class of IFNs and are associated with natural clearance of HCV, by being directly produced in response to the infection. IFN-*λ* genes are clustered on chromosome 19 and encode IFN-*λ*1 (*IL29*), IFN-*λ*2 (*IL28A*), and IFN-*λ*3 (*IL28B*) [12, 13]. The lambda-IFNs have a restricted target cell range and they signal via the JAK/STAT pathway, upregulating the expression of a set of interferon-stimulated genes (ISGs), through which they inhibit virus replication [14, 15].

Thus far, not many studies have looked into the correlation of IL28B serum levels with the different IL28B genetic variants in HCV patients [16], and there are few reports analyzing the significance of these variants in the progression of HCV infected patients to advanced stages of the disease. Therefore, the current study aims to determine the correlation of serum IL-28 levels in relation to IL-28B genetic variations, different viral genotypes/subgenotypes, and relation to disease progression. Furthermore, the study aims at understanding the impact of IL28B SNPs in the development of HCV-related cirrhosis and HCC.

## 2. Materials and Methods

### 2.1. Patients

This study was conducted at Riyadh area of Saudi Arabia where samples were collected from three major hospitals, namely, King Faisal Specialist Hospital & Research Center, King Khalid University Hospital and Riyadh Military Hospital. The study was approved by the ethics committees from each hospital and conformed to the ethical guidelines of the 2000 declaration of Helsinki. All participating patients signed an informed consent prior to enrollment in the study. The study included 678 patients who were divided into three groups according to their disease progression including chronic (group 1), cirrhosis (group 2), and cirrhosis+HCC (group 3). Group 1: chronically infected patients (detectable HCV RNA >6 months) without cirrhosis or HCC. Patients in this group did not have any clinical or biochemical evidence of cirrhosis and had a fibroscan that shows less than stage 2 fibrosis. Group 2: patients with cirrhosis who are diagnosed based on either liver biopsy or clinical conditions including the presence of the following criteria; platelet count <90,000/L, radiological (ultrasonography [US] or computed tomography [CT]) evidence of cirrhosis, or esophageal varices (demonstrated by endoscopy), and signs of liver dysfunction including; albumin level <30 g/L, INR ≥1.5, or bilirubin level >35 *μ*mol/L. Group 3: patients with HCC on the background of cirrhosis. The diagnosis of HCC was based upon established CT or magnetic resonance imaging (MRI) criteria in published guidelines for the diagnosis and management of HCC [17]. Enhancement of a liver lesion during the arterial phase and contrast washout during the portal phase, in patients with background cirrhosis was considered diagnostic of HCC. Trucut biopsy or fine needle aspiration was obtained only where considerable doubt existed after imaging studies and alpha-fetoprotein (AFP) tests. The study also included 600 uninfected healthy subjects, used as a control group, who were characterized by the absence of any known serological marker for HCV, HCV RNA or any evidence of liver disease.

### 2.2. Subgenotyping of HCV

HCV RNA was extracted from sera using QIAmp MinElute Virus Spin Kit (QIAGEN, Santa Clarita, CA, USA) following manufacturer's recommendations. Extracted RNA was converted to cDNA as described before by Al-Qahtani et al. [18] and subgenotypes were determined according to the procedures described by Murphy et al. [19]. PCR products were sequenced using BigDye Terminator v3.1 cycle sequencing Kit (Applied Biosystems, Foster City, CA, USA) following manufacturer's recommendations. DNA sequence was then analyzed using BLASTN 2.2.23+ software (http://blast.ncbi.nlm.nih.gov/blast.cgi) against all sequences in the database to determine the subgenotype of each sample. The sequence that showed the lowest *e*-value and maximum identity was taken as the subgenotype of the sample analyzed.

### 2.3. Detection of Serum IL-28B by ELISA

IL-28B concentration was determined in patients' sera using a sandwich ELISA according to the manufacturer's recommendations (USCNK, Life Science Inc., Wuhan, China). Briefly, sera or recombinant human IL-28B, as standards, were added to plates coated with a monoclonal antibody against IL-28B and incubated at room temperature for two hours. All analyses and calibrations were carried out in a duplicate. Optical density was determined using a microtiter plate reader (BioTek ELx50, Winooski, VT, USA) at 450 nm with the lower end of sensitivity of the assay was 15.6 pg/mL and the upper limit was 1000 pg/mL. Samples with readings greater than the upper limit were diluted until their absorbance readings fall within the linear range of the assay.

### 2.4. Statistical Analysis

Statistical analyses were completed using SPSS v. 17.0 (SPSS Inc., Chicago, IL, USA). Chi-square test was used to compare the genotypic distribution of IL28 SNPs among patients and controls. The association results were expressed in terms of odds ratio (OR) and their 95% confidence intervals (CI). A *P* value of <0.05 was considered to be statistically significant. The SNPs were tested for Hardy-Weinberg equilibrium (HWE) using the DeFinetti program (http://ihg.gsf.de/cgi-bin/hw/hwa1.pl). All correlation analyses were performed in the R software environment (http://www.r-project.org/).

## 3. Results

### 3.1. Association of IL28B Polymorphisms with HCV Infection

Five SNPs in the vicinity of the IL28B gene (rs8105790, rs8099917, rs7248668, rs12979860, and rs12980275) were genotyped in 678 HCV-infected patients and 600 healthy, uninfected control subjects. The demographic and clinical data are shown in Table 1. There were significant differences between the patient groups in all categories except for viral load, ALT, and creatinine levels.

When the patient group was compared to the uninfected control subjects, all SNPs were found to have a significant association in relation to HCV infection (Table 2). The risk allele “G” for the SNP rs8099917 was found to be significant when the patient group was compared to the uninfected control group with an OR of 2.55 (95% C.I. 2.062–3.160), *χ*²-value of 77.16, and *P* < 0.0001. Under the dominant model, a significant association was observed with an OR of 2.047 (95% C.I. 1.593–2.630), *χ*²-value of 31.91, and *P* < 0.0001. While under the recessive model, a significant association was observed with an OR of 0.132 (0.075–0.233), *χ*²-value of 64.25, and *P* < 0.0001, indicating that inheriting a homozygous GG genotype would increase the risk of HCV infection by nearly 3 times than those carrying the heterozygous GT genotype. Likewise, a similar significant result was obtained for rs8105790 under the recessive model, with an OR of 0.089 (0.053–0.152), *χ*²-value of 116.35, and *P* < 0.0001, indicating that patients who are homozygous to CC genotype would have nearly 5 times increase in their risk of HCV infection than those carrying the heterozygous CT genotype.

Haplotype analysis revealed three blocks that were significantly distributed between patient group and uninfected healthy control subjects. The blocks were for SNPs rs12980275 and rs12979860, respectively, AC (freq. = 0.603, *χ*² = 19.06, *P* < 0.0001), GT (freq. = 0.256, *χ*² = 50.08, *P* < 0.0001), and AT (freq. = 0.082, *χ*² = 11.055, *P* = 0.0009) (Table 3).

### 3.2. Association of IL28B Polymorphisms with Disease Progression

In order to determine whether these SNPs play any role in the progression of the HCV infection to liver cirrhosis and HCC, further analysis was done by comparing HCV-infected patients suffering from cirrhosis (group 2) to patients with chronic HCV infection (group 1) and by comparing patients who have progressed to HCC as a result of persistent HCV infection (group 3) to group 1 patients. No significant observations were found for any of the five SNPs in both comparisons (data not shown). Furthermore, these SNPs were also analyzed by comparing groups 2+3 combined against group 1, and none of the SNPs showed any significant associations (Table 4).

### 3.3. Correlation between IL28B Serum Level and HCV Infection

**Figure 1:**
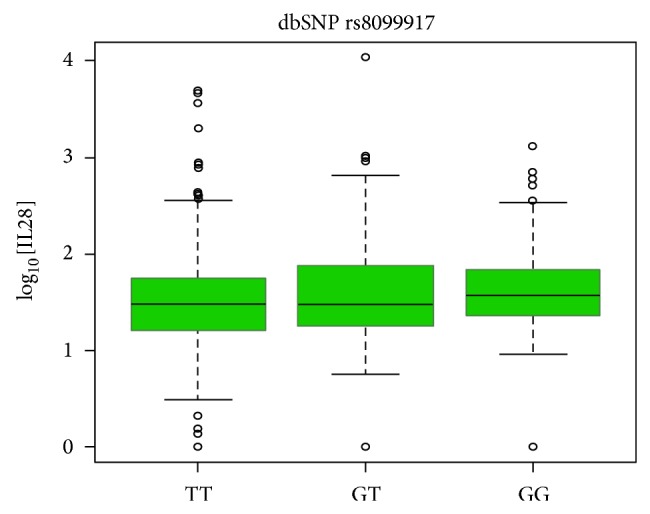
Boxplot analysis of rs8099917 genotypes against average serum IL28B concentration.

Next, to evaluate if there is a correlation between serum IL28B levels and the different SNP genotypes, a box-plot analysis was performed for the different SNPs against average logarithmic values of IL28B levels. Only rs8099917 showed a significant correlation in relation to IL28B serum levels. The SNP rs8099917 showed an increase of ~0.06 in log_10_ of serum IL28B levels, with the addition of each G-allele, suggesting that HCV-infected patients with GG genotype have higher serum IL28B levels (Figure 1 and Table 5).

**Figure 2:**
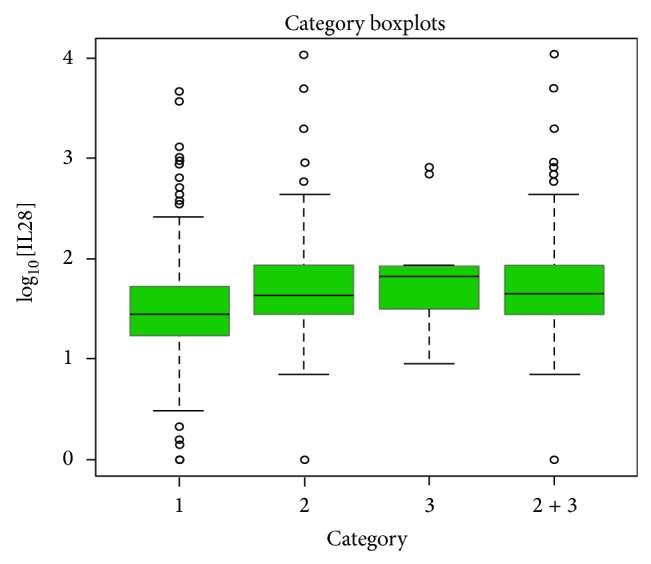
Boxplot analysis of average serum IL28B concentration with different outcomes of HCV infection.

A similar analysis was done to estimate the correlation of serum IL28B levels with regard to different groups of patients classified according to their severity of the disease. A significant result was obtained when group 2 and groups 2+3 were compared to group 1 with an increase of 0.16 in log_10_ of IL28B serum level for group 2 and group 2+3 samples, while no additional significant effect was observed for group 3 alone (Figure 2 and Table 6).

Based on a correlation of average log_10_ of serum IL28 levels, no significant differences were observed between the different HCV genotypes/subgenotypes (classified based on partial NS5B sequence) and serum IL28 levels (Tables 7 and 8).

A linear regression analysis was performed to determine if a significant relationship exists between the different parameters (such as IL28B SNPs, viral subgenotypes, and patient category) and serum IL28 levels (Table 7). No significant relationship was observed either for the IL28B genotypes or the viral subgenotypes, with respect to IL28 levels, while a significant relationship was observed when the different patient groups were analyzed in relation to IL28 levels (for group 2 — *r* ^2^ = 0.245, *P* = 0.0002; for group 3 — *r* ^2^ = 0.337; *P* = 0.045; for group 2+3, *r* ^2^ = 0.255; *P* < 0.0001), suggesting that the advancement of HCV patients to different stages may depend on the serum IL28B levels.

To confirm the results obtained in Table 7, a robust linear regression, by excluding all the outlier values, was performed. This is to determine if the linear regression model failed as a result of these nonconformal outliers or whether no true relationship existed between the various parameters and serum IL28B levels (Table 8). The GG genotype of rs8099917 was found to have a significant relation with serum IL28B levels in the robust model (*P* value = 0.043, regression coefficient, *r* ^2^ = 0.115). A significant relationship was also observed for group 2 and group 2+3 (*r* ^2^ = 0.161 and 0.156, *P* value = 0.005 and 0.003, resp.).

### 3.4. Receiver Operating Characteristic (ROC) Curve Analysis

**Figure 3:**
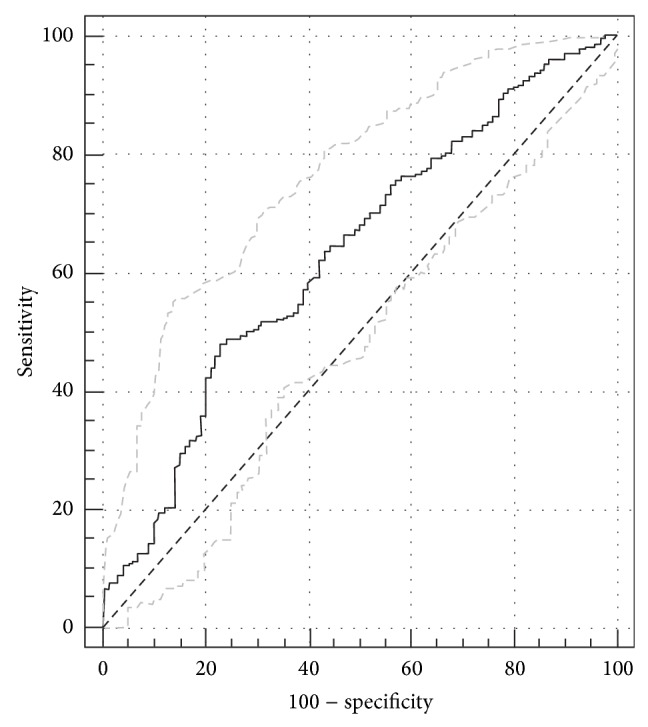
ROC curve of the true positive rate (sensitivity), false positive rate (100-specificity) predictive values of the IL28B serum level concentration among patients infected with HCV and individual with liver cirrhosis+HCC patients.

ROC analysis was used to establish the specificity and sensitivity of using IL28B serum concentration as a marker for HCV-associated diseases. The best cut-off values were 75.3% sensitivity and 38.5% specificity when chronic patients (group 1) were compared with cirrhosis+HCC patients (groups 2+3). The area under the curve was 0.60 (95% Confidence interval: 0.55–0.65, and *P* = 0.005). The cut-off value of IL-28B in the serum that could distinguish chronic patients from Cirr+HCC patients was 51.0 pg/mL (Figure 3).

## 4. Discussion

IL-28B belongs to type III IFNs, also called IFN-*λ*s, which was discovered in 2002 by two independent groups [12, 13]. Though structurally different from Type-1 IFNs, the IFN-*λ*s exhibit a similar mechanism as that of IFN-*α*'s in terms of their signaling and biological activities. Although both groups of IFNs play a major role in antiviral activities, IFN-*λ*s have recently generated much interest because of their link to the spontaneous resolution and successful treatment of HCV infection [20].

Recent studies have reported that the mRNA expression levels of IL28 in peripheral blood mononuclear cells are lower in patients undergoing treatment and who carry the minor G allele for the SNP rs8099917. This seems theoretically reasonable, as increased levels of IL28B together with the combination therapy would facilitate clearance of the virus [9, 10].

On the contrary, Abe et al. reported that the expression levels of IL28B in liver are lower in PEG-IFN-treated patients having rs8099917 TT genotype [21]. The present study is in agreement with the findings of Abe et al., as the serum IL28B levels were found to decrease with the addition of each T-allele in HCV-infected patients [21]. A plausible explanation of increased expression of IL28B levels in patients undergoing treatment could be for the unique capability of IFN-*λ*s to increase its expression when induced by IFN-*α*, that is, for patients being treated with PEG-IFN *α*/*β*-ribavirin would most likely have increased levels of IL28B levels as a result of its direct stimulation with IFN-*α* [22, 23]. Further investigations are needed to understand how the serum IL28B levels vary according to the IL28B genetic polymorphisms, since such polymorphisms could affect expression and/or the stability of IL28B mRNA. Also, it is not clear at what level is the expression of IL28B being affected, that is, whether at the transcription and/or the translation levels in HCV-infected patients undergoing treatment with PEG-IFN *α*/*β*-ribavirin.

In the current study, rs8099917 was the only SNP that significantly correlated with the IL28B serum levels, while the other four SNPs, including rs12979860, failed to show any correlation with serum IL28B levels. This finding is at variance with the results obtained by Langhans et al., as the study showed correlation of serum level with the C allele of rs12979860 SNP [16]. However, it is unclear if rs8099917 variants were tested in this study as no data were shown. We also observed a significant relationship between IL28B levels and advanced stages of HCV infection as IL28 serum level tends to increase with the severity of the disease; however, further investigations are needed to explain the effect of IL28B levels on disease progression.

IL28B variants have been reported to have a significant effect on fibrosis progression in HCV-infected patients [24, 25]. Recently, a study conducted on Italian population, reported that the HCV-related cirrhotic patients carrying rs12979860-C allele are less likely to undergo liver transplantation or suffer from end-stage liver diseases such as HCC [26]. A similar finding was reported that the C-allele has a protective role in the development of HCV-induced HCC and in HCV recurrence after liver transplantation [27]. On the contrary, other studies have reported that the variants of IL28B may not be involved in the hepatocarcinogenesis of HCV-infected patients [25, 28]. Results reported in the present study were in agreement with that of the latter studies as none of the five IL28B genetic variants investigated in this study had any significant effect on the advancement of HCV infection to cirrhosis and/or HCC; though, we did observe that these SNPs could have a significant role in the establishment of HCV infection as they showed significant differences in distribution between patients and healthy control subjects. However, our results must be interpreted with caution in view of the small number of HCC patients included in the study. Therefore, studies with larger number of cirrhotic and HCC patients are needed to ascertain the role of IL28B polymorphisms in hepatocarcinogenesis among HCV-infected patients.

Although there are several studies reporting the association of HCV virus genotype with treatment response, there are no reports that correlate IL28B serum levels with HCV viral genotypes/subgenotypes. This is the first study that has looked into such a correlation, but, surprisingly, our findings indicate that there is no correlation between IL28B serum level and genotypes/subgenotypes of HCV. This is consistent with other studies that examined serum level of cytokines or chemokines in relation to HCV genotypes [29, 30]. These studies indicated that no correlation was found between HCV genotypes, cytokine/chemokine serum levels, and disease progression or response to treatment in HCV-infected patients.

Furthermore, we have also found that the IL28B serum levels have a significant correlation with the different outcomes of HCV infection, especially among patients suffering from cirrhosis and HCC, where the IL28 serum levels tend to increase with progression of the disease. However, it is unclear why the IL28 serum levels are affected by the different outcomes of HCV infection and by rs8099917-G allele, and therefore additional research is required to establish these correlations.

The analysis of ROC curve revealed the usefulness of the IL28B serum level as a prognostic marker for disease progression in HCV-infected patients. It was possible to differentiate patients with liver complication, such as liver cirrhosis and HCC, from chronic patients. However, these results must be carefully interpreted as the area under the curve was not very high (0.6) and this could be attributed to the low number of patients with cirrhosis and HCC.

## 5. Conclusions

The T-allele of rs8099917 was found to be correlated with reduced IL28B serum levels. Also, IL28B variants might play a significant role in HCV infection, but they may not be considered as risk factors in the progression of HCV infection to advanced stages such as liver cirrhosis and HCC.

## Figures and Tables

**Table 1:** Basic characteristics of all subjects included in this study.

Variable	Chronic HCV	Cirrhosis+HCC	Control	*P* value
Age (yrs.)^¶^	48.00 (36.00–57.00)	57.00 (48.00–63.00)	29.00 (24.00–36.00)	<0.000^§^
Sex				
Male count (%)	274 (50.5%)	94 (51.4%)	568 (94.7%)	<0.000^*ϕ*^
Female count (%)	269 (49.5%)	89 (48.6%)	32 (5.3%)	
BMI^¶^	31.88 (26.53–71.43)	28.72 (24.57–34.13)		0.014^§^
Platelet count (10^9^/L)^¶^	216.00 (169.00–284.50)	148.00 (102.50–200.50)		<0.000^§^
Bil (umol/L)^*^	13.21 ± 26.18	25.07 ± 73.34		<0.000^*ψ*^
ALT (IU/L)^*^	74.53 ± 66.9	83.66 ± 62.47		0.766^*ψ*^
AST (IU/L)^*^	45.07 ± 40.299	75.16 ± 57.16		<0.000^*ψ*^
AFP (ug/L)^*^	6.36 ± 22.03	15.97 ± 27.14		<0.000^*ψ*^
ALP (IU/L)^*^	106.30 ± 53.07	122.65 ± 76.77		0.004^*ψ*^
Creatinine (umol/L)^*^	110 ± 151.31	97.72 ± 127.30		0.074^*ψ*^
HCV load IU/mL (log⁡10)^¶^	5.80 (4.788–6.41)	6.025 (5.49–6.44)		0.511^§^
HCV genotypes				
4 (357)	249 (70%)	108 (30%)		<0.0001
1 (148)	115 (77.7%)	33 (22.3%)		
Others^¶¶^ (173)	154 (89%)	19 (11%)		

BMI: body mass index; Bil: bilirubin; ALT: alanine aminotransferase; AST: aspartate aminotransferase; AFP: *α*-fetoprotein, ALP: alkaline phosphatase. ^*^Values are expressed as mean ± SD, ^¶^values are expressed as median-interquartile range (25th–75th), ^¶¶^includes genotypes 2, 3, and untypable genotypes. ^§^Nonparametric tests, ^*ϕ*^Chi-squared tests, and ^*ψ*^independent *t*-tests.

**Table 2:** Genotypic distribution for IL-28B gene polymorphism when patient group (groups “1+2+3”) was compared to control group.

SNPs	Genotype/allele distribution	Healthy controls *n* = 600	HCV patients *n* = 678	OR (95% C.I.)	*χ* ^2^	*P* value
rs8105790				2.784 (2.340–3.311)	137.99	**<0.0001**
	CC	16 (2.7%)	159 (23.4%)			
	CT	233 (38.8%)	280 (41.3%)			
	TT	351 (58.5%)	239 (35.3%)			
	**C**	265 (22.1%)	598 (44.1%)			
	T	935 (77.9%)	758 (55.9%)			
	CC + CT versus TT			2.589 (2.065–3.247)	69.23	**<0.0001**
	CC versus CT + TT			0.089 (0.053–0.152)	116.35	**<0.0001**

rs8099917				2.552 (2.062–3.160)	77.16	**<0.0001**
	GG	14 (2.3%)	104 (15.3%)			
	GT	114 (19.0%)	138 (20.4%)			
	TT	472 (78.7%)	436 (64.3%)			
	**G**	142 (11.8%)	346 (25.5%)			
	T	1058 (88.2%)	1010 (74.5%)			
	GG + GT versus TT			2.047 (1.593–2.630)	31.91	**<0.0001**
	GG versus GT + TT			0.132 (0.075–0.233)	64.25	**<0.0001**

rs7248668				1.863 (1.533–2.264)	39.72	**<0.0001**
	AA	58 (9.7%)	94 (13.9%)			
	AG	79 (13.2%)	172 (25.4%)			
	GG	463 (77.2%)	412 (60.7%)			
	**A**	195 (16.3%)	360 (26.5%)			
	G	1005 (83.8%)	996 (73.5%)			
	AA + AG versus GG			2.182 (1.708–2.788)	39.65	**<0.0001**
	AA versus AG + GG			0.665 (0.470–0.941)	5.35	**0.0206**

rs12979860				1.476 (1.250–1.743)	21.15	**<0.0001**
	TT	59 (9.9%)	73 (10.8%)			
	CT	230 (38.6%)	367 (54.1%)			
	CC	307 (51.5%)	238 (35.1%)			
	**T**	348 (29.2%)	513 (37.8%)			
	C	844 (70.8%)	843 (62.2%)			
	TT + CT versus CC			1.964 (1.568–2.460)	34.88	**<0.0001**
	TT versus CT + CC			0.911 (0.634–1.308)	0.26	0.61213

rs12980275				1.765 (1.488–2.093)	42.97	**<0.0001**
	GG	59 (9.8%)	89 (13.12%)			
	AG	184 (30.7%)	327 (48.2%)			
	AA	357 (59.5%)	262 (38.6%)			
	**G**	302 (25.2%)	505 (37.2%)			
	A	898 (74.8%)	851 (62.8%)			
	GG + AG versus AA			2.333 (1.863–2.920)	55.44	**<0.0001**
	GG versus AG + AA			0.722 (0.509–1.023)	3.37	0.06631

Risk allele marked in BOLD letter.

**Table 3:** Haplotype analysis for SNPs rs12980275 and rs12979860 when patient group (groups “1+2+3”) was compared to control group.

Haplotype	Freq.	HCV patients, healthy control ratio counts	HCV, healthy control frequencies	Chi-square	*P* value
Block 1					
AC	0.603	763.3 : 592.7, 777.1 : 422.9	0.563, 0.648	19.064	1.26 × 10^−5^
GT	0.256	425.3 : 930.7, 229.5 : 970.5	0.314, 0.191	50.086	1.47 × 10^−12^
AT	0.082	87.7 : 1268.3, 120.9 : 1079.1	0.065, 0.101	11.055	9 × 10^−4^
GC	0.06	79.7 : 1276.3, 72.5 : 1127.5	0.059, 0.060	0.032	0.857

**Table 4:** Genotypic distribution for IL-28B gene polymorphism when chronic HCV patients (group 1) were compared to individuals with liver cirrhosis and HCC carriers (groups “2+3”).

SNPs	Genotype/allele distribution	(Chronic HCV) *n* = 518	(Cirr + HCC) *n* = 160	OR (95% C.I.)	*χ* ^²^	*P* value
rs8105790				1.049 (0.815–1.350)	0.14	0.710
	CC	119 (23%)	40 (25%)			
	CT	216 (42%)	64 (40%)			
	TT	183 (35%)	56 (35%)			
	**C**	454 (43.8%)	144 (45%)			
	T	582 (56.2%)	176 (55%)			
	CC + CT versus TT			1.014 (0.700–1.471)	0.01	0.939
	CC versus CT + TT			0.895 (0.592–1.351)	0.28	0.596

rs8099917				0.986 (0.739–1.315)	0.01	0.923
	GG	80 (15.4%)	24 (15%)			
	GT	105 (20.3%)	33 (20.6%)			
	TT	333 (64.3%)	103 (64.4%)			
	**G**	265 (25.6%)	81 (25.3%)			
	T	771 (74.4%)	239 (74.7%)			
	GG + GT versus TT			0.996 (0.688–1.442)	0	0.983
	GG versus GT + TT			1.035 (0.631–1.698)	0.02	0.891

rs7248668				1.204 (0.912–1.590)	1.72	0.190
	AA	65 (12.5%)	29 (18.1%)			
	AG	136 (26.3%)	36 (22.5%)			
	GG	317 (61.2%)	95 (59.4%)			
	**A**	266 (25.7%)	94 (29.4%)			
	G	770 (74.3%)	226 (70.6%)			
	AA + AG versus GG			1.079 (0.752–1.549)	0.17	0.679
	AA versus AG + GG			0.648 (0.402–1.046)	3.18	0.074

rs12979860				1.167 (0.903–1.507)	1.39	0.238
	TT	50 (9.7%)	23 (14.4%)			
	CT	283 (54.6%)	84 (52.5%)			
	CC	185 (35.7%)	53 (33.1%)			
	**T**	383 (37%)	130 (40.6%)			
	C	653 (63%)	190 (59.4%)			
	TT + CT versus CC			1.122 (0.771–1.632)	0.36	0.548
	TT versus CT + CC			0.636 (0.375–1.080)	2.84	0.092

rs12980275				0.929 (0.716–1.206)	0.3	0.580
	GG	69 (13%)	20 (12.5%)			
	AG	252 (49%)	75 (46.9%)			
	AA	197 (38%)	65 (40.6%)			
	**G**	390 (38%)	115 (36%)			
	A	646 (62%)	205 (64%)			
	GG + AG versus AA			0.897 (0.625–1.288)	0.35	0.555
	GG versus AG + AA			1.076 (0.631–1.832)	0.07	0.788

Risk allele marked in BOLD letter.

**Table 5:** Correlation of average IL28B serum levels with the genotypes of rs8099917.

	Value	Std. error	*t*-value	*P* value
(Intercept)	1.586	0.025	58.82	0
rs8099917-GT	0.057	0.049	1.16	0.25
rs8099917-GG	0.115	0.057	2.03	**0.04**

**Note:** results for “intercept” correspond to “GG” genotype; that is, log⁡10 (conc.) = 1.586 for TT genotype, with ~0.06 increase in log⁡10 (conc.) with each additional G-allele.

**Table 6:** Correlation of average IL28B serum levels with different outcomes of HCV infection.

	Value	Std. error	*t*-value	*P* value
(Intercept)	1.461	0.027	54.77	0
Group 2	0.161	0.057	2.82	**0.005**
Group 3	0.126	0.161	0.79	0.43
Groups 2 + 3	0.156	0.053	2.95	**0.003**

**Note:** results for “intercept” correspond to group = 1; that is, log⁡10 (conc.) = 1.46 for group 1 samples, with ~0.16 increase in log⁡10 (conc.) for group 2 samples, and no additional sig. affect in log⁡10 (conc.) for group 3 samples.

**Table 7:** Linear regression of serum IL28B levels with different factors.

Factor	Value	Coefficient	*P* value
rs12979860	CT	0.033	0.525
rs12979860	TT	0.043	0.593
rs12980275	AG	0.059	0.251
rs12980275	GG	0.021	0.769
rs8105790	CT	0.011	0.838
rs8105790	CC	−0.029	0.644
rs8099917	GT	0.098	0.0915
rs8099917	GG	0.107	0.111
rs7248668	AG	0.097	0.0889
rs7248668	AA	0.034	0.602
HCV genotype	1a	0.269	0.618
HCV genotype	1b	0.285	0.595
HCV genotype	1c	−0.176	0.815
HCV genotype	1g	0.12	0.845
HCV genotype	2	0.318	0.603
HCV genotype	2a	0.217	0.738
HCV genotype	2c	0.127	0.827
HCV genotype	2k/1b	−0.187	0.774
HCV genotype	3	−0.097	0.881
HCV genotype	3a	0.198	0.721
HCV genotype	4	0.034	0.954
HCV genotype	4 beta	−0.244	0.745
HCV genotype	4a	0.214	0.688
HCV genotype	4d	0.272	0.609
HCV genotype	4l	0.229	0.76
HCV genotype	4m	−0.219	0.736
HCV genotype	4n	0.325	0.567
HCV genotype	4o	0.44	0.472
HCV genotype	4o/4 beta	0.034	0.956
HCV genotype	4r	0.366	0.502
All versus 1a		0.028	0.787
All versus 1b		0.049	0.521
All versus 1a/b		0.047	0.472
All versus 4a		−0.042	0.472
All versus 4d		0.042	0.482
All versus 4a/d		−0.001	0.985
Group 2		0.245	**0.0002**
Group 3		0.337	**0.044**
Groups 2 + 3		0.255	**0.00005**

**Table 8:** Robust regression of serum IL28B levels with different factors.

Factor	Value	Coefficient	*P* value
rs12979860	CT	0.050	0.269
rs12979860	TT	0.032	0.645
rs12980275	AG	0.038	0.394
rs12980275	GG	0.010	0.87
rs8105790	CT	0.041	0.391
rs8105790	CC	0.032	0.556
rs8099917	GT	0.057	0.246
**rs8099917**	**GG**	**0.115**	**0.043**
rs7248668	AG	0.051	0.3
rs7248668	AA	0.038	0.49
HCV genotype	1a	0.311	0.854
HCV genotype	1b	0.219	0.897
HCV genotype	1c	−0.176	0.666
HCV genotype	1g	−0.400	0.818
HCV genotype	2	0.318	0.853
HCV genotype	2a	0.217	0.9
HCV genotype	2c	0.127	0.941
HCV genotype	2k/1b	−0.187	0.914
HCV genotype	3	−0.097	0.955
HCV genotype	3a	0.198	0.907
HCV genotype	4	0.489	0.776
HCV genotype	4 beta	−0.244	0.551
HCV genotype	4a	0.152	0.928
HCV genotype	4d	0.184	0.913
HCV genotype	4l	0.229	0.575
HCV genotype	4m	−0.219	0.9
HCV genotype	4n	0.325	0.848
HCV genotype	4o	0.006	0.997
HCV genotype	4o/4 beta	−0.189	0.995
HCV genotype	4r	0.259	0.879
All versus 1a		0.141	0.0938
All versus 1b		0.042	0.507
All versus 1a/b		0.089	0.096
All versus 4a		−0.045	0.363
All versus 4d		0.005	0.925
All versus 4a/d		−0.039	0.397
Group 2		0.161	**0.005**
Group 3		0.126	0.432
Groups 2 + 3		0.156	**0.003**
